# Differential Involvement of Brain-Derived Neurotrophic Factor in Reconsolidation and Consolidation of Conditioned Taste Aversion Memory

**DOI:** 10.1371/journal.pone.0049942

**Published:** 2012-11-21

**Authors:** Yue Wang, Tian-Yi Zhang, Jian Xin, Ting Li, Hui Yu, Na Li, Zhe-Yu Chen

**Affiliations:** Department of Neurobiology, Shandong Provincial Key Laboratory of Mental Disorders, School of Medicine, Shandong University, Jinan, Shandong, People’s Republic of China; Prince Henry’s Institute, Australia

## Abstract

Consolidated memory can re-enter states of transient instability following reactivation, which is referred to as reconsolidation, and the exact molecular mechanisms underlying this process remain unexplored. Brain-derived neurotrophic factor (BDNF) plays a critical role in synaptic plasticity and memory processes. We have recently observed that BDNF signaling in the central nuclei of the amygdala (CeA) and insular cortex (IC) was involved in the consolidation of conditioned taste aversion (CTA) memory. However, whether BDNF in the CeA or IC is required for memory reconsolidation is still unclear. In the present study, using a CTA memory paradigm, we observed increased BDNF expression in the IC but not in the CeA during CTA reconsolidation. We further determined that BDNF synthesis and signaling in the IC but not in the CeA was required for memory reconsolidation. The differential, spatial-specific roles of BDNF in memory consolidation and reconsolidation suggest that dissociative molecular mechanisms underlie reconsolidation and consolidation, which might provide novel targets for manipulating newly encoded and reactivated memories without causing universal amnesia.

## Introduction

The traditional theories of how the brain forms new memories consider that a consolidation process fixes initially fragile memories over time until they undergo ‘stabilization’ in the brain. Once consolidated, the disruption of these memories becomes difficult [1]. However, other data challenge this claim, indicating that the retrieval of memory traces can induce an additional labile phase that requires an active process to stabilize memory after retrieval [2], [3], [4]. This process has been referred to as reconsolidation and has recently been considered an important component of long-term memory processing [5], [6], [7]. The study of reconsolidation has been extended to numerous species, including humans [8], [9] and rodents [10], [11], across a broad range of learning tests, using a variety of manipulations to block memory [5], [7]. Although there is much support for the generality of reconsolidation, the exact molecular mechanisms underlying this process remain unexplored [7]. Investigating the detailed molecular mechanisms involved in reconsolidation is important not only to further understand the process of memory but also for future clinical therapy.

Brain-derived neurotrophic factor (BDNF) is a small dimeric protein that regulates neuronal survival and differentiation and plays a critical role in synaptic plasticity and memory processes [12], [13], [14]. Increasing evidence has indicated that BDNF signaling via the tropomyosin-related kinase receptor B (TrkB) in the hippocampus or amygdala contributes to spatial or fear memory consolidation [10], [15], [16], [17], [18]. Moreover, we have recently observed that BDNF synthesis in the central nuclei of the amygdala (CeA) and insular cortex (IC) is involved in the consolidation of conditioned taste aversion (CTA) memory [19]. However, whether BDNF synthesis in the CeA or IC is required for memory reconsolidation is still unclear, as reconsolidation and consolidation might rely on different molecular and cellular processes. For example, in contextual fear memory, hippocampal BDNF is involved in consolidation but not reconsolidation [10].

CTA memory is associative hippocampus-independent cortical learning that can be obtained after a single trial and persists for long time [20], which make CTA a useful model to study the different phases of memory, including reconsolidation [21]. In the present study, using loss-of-function and gain-of-function approaches, we demonstrated that although BDNF synthesis in both the IC and CeA is involved in CTA consolidation, BDNF in the IC but not in the CeA is required for CTA reconsolidation.

**Figure 1:**
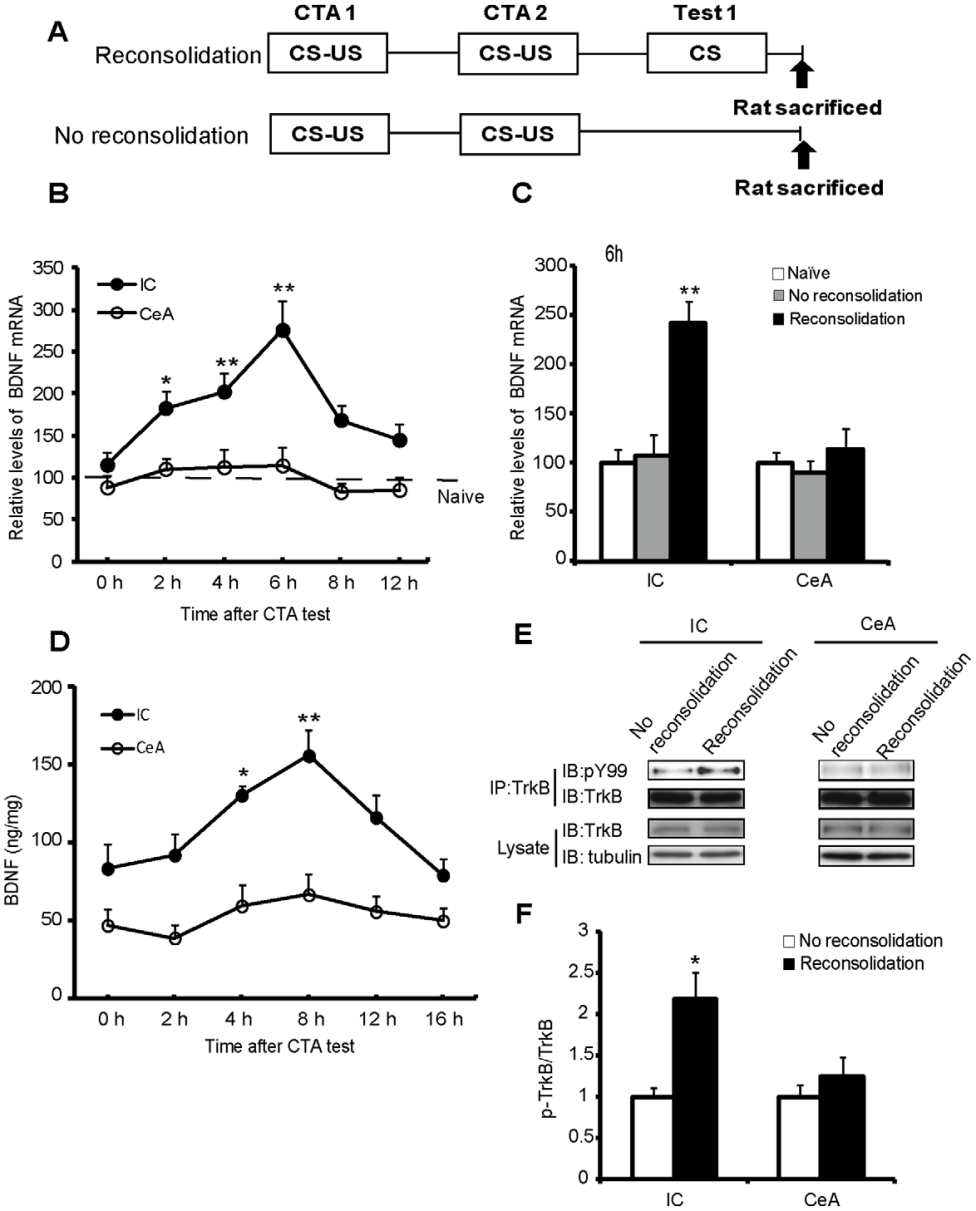
Temporal and spatial changes of BDNF during CTA reconsolidation. (A) Schematic of the experimental design for sample collection. (B) Temporal and spatial changes in BDNF mRNA expression during CTA reconsolidation. The relative levels of BDNF mRNA expression in the IC and CeA were normalized to the levels of the naïve group. n = 5–6 per time point; *P<0.05, **P<0.01, compared with the values at the 0 h time point in the two respective brain regions. (C) The increased BDNF mRNA levels in the IC were induced specifically through reconsolidation. n = 5 per group. **P<0.01, compared with the values in the IC of the naïve group. (D) Temporal and spatial changes in the BDNF protein levels during CTA reconsolidation. n = 5 per time point. *P<0.05, **P<0.01, compared with the values at the 0 h time point in the two respective brain regions. (E, F) The spatial changes in the p-TrkB levels during CTA reconsolidation. The samples were obtained at 4 h post retrieval. The quantification of immunoblotting in (E) is shown as the percentage of p-TrkB receptor relative to the total TrkB obtained from immunoprecipitation, which was normalized to the no reconsolidation group. n = 3 per group. **P<0.01, compared with the values of the no reconsolidation group. All values are presented as the mean ± S.E.M.

## Materials and Methods

### Animals

Adult male Wistar rats (2 months old, weighing 250–300 g), obtained from Vital River Laboratories (Beijing, China), were used for the experiments. All rats were housed individually at 22±2°C with a 12 h light/dark cycle and *ad libitum* access to food and water unless otherwise indicated. The study was approved by the ethics committee of the School of Medicine of Shandong University. All procedures were approved by the Institutional Animal Care and Use Committee (IACUC) of Shandong University. All surgeries were performed with chloral hydrate, and all efforts were made to alleviate suffering.

### Behavioral Procedures

The associative learning paradigm was known as CTA, and the consolidation and reconsolidation procedures were performed as described in previous reports [21], [22] with minor modifications. In the CTA paradigm, saccharin (0.1%, w/v) was used as the novel taste (conditioned stimulus, CS), while intraperitoneal (i.p.) injection of LiCl (0.15 M, 2% body weight per rat) was used as a malaise-inducing agent (unconditioned stimulus, US). In advance of the CTA procedure, 24 h of water deprivation was required, and the rats would get their daily water ration of two pipettes of water (each containing 10 mL) for 10 min over the next 3–5 consecutive days. On the conditioning day, the rats were allowed to drink saccharin instead of water for 10 min. Forty minutes after drinking, an i.p. injection of LiCl was administered. On each of the next two days, the rats were again presented with water for 10 min; the test day occurred 72 h after conditioning. Six pipettes (three containing 5 mL of water and three containing 5 mL of saccharin) were randomly ordered and offered to the rats as a multiple-choice test for 10 min. During these 10 min, the rats had free access to either water or saccharin. Described above is the original CTA procedure with only one conditioning [23] (see Figure S1). An additional CTA training was performed before the reconsolidation process according to Eisenberg et al. [21]. Namely, 24 h after the first CTA conditioning, a second CTA training was performed; 96 h after the second CS-US pairing, a multiple-choice test was used to determine the degree of aversion. Subsequently, the process of reconsolidation was initiated (see Figure S1). For weak CTA conditioning, all rats were trained as above, except that on the conditioning day, the rats received 0.075 M LiCl (i.p. 2% body weight) instead of 0.15 M LiCl. The result was evaluated by an aversion index (AI), which was defined as AI = water consumed/(water+saccharin consumed)×100%. Usually, an AI>50% indicates a higher preference for water over saccharin. The second and third tests were performed 24 h and 48 h after the first test, respectively.

**Figure 2:**
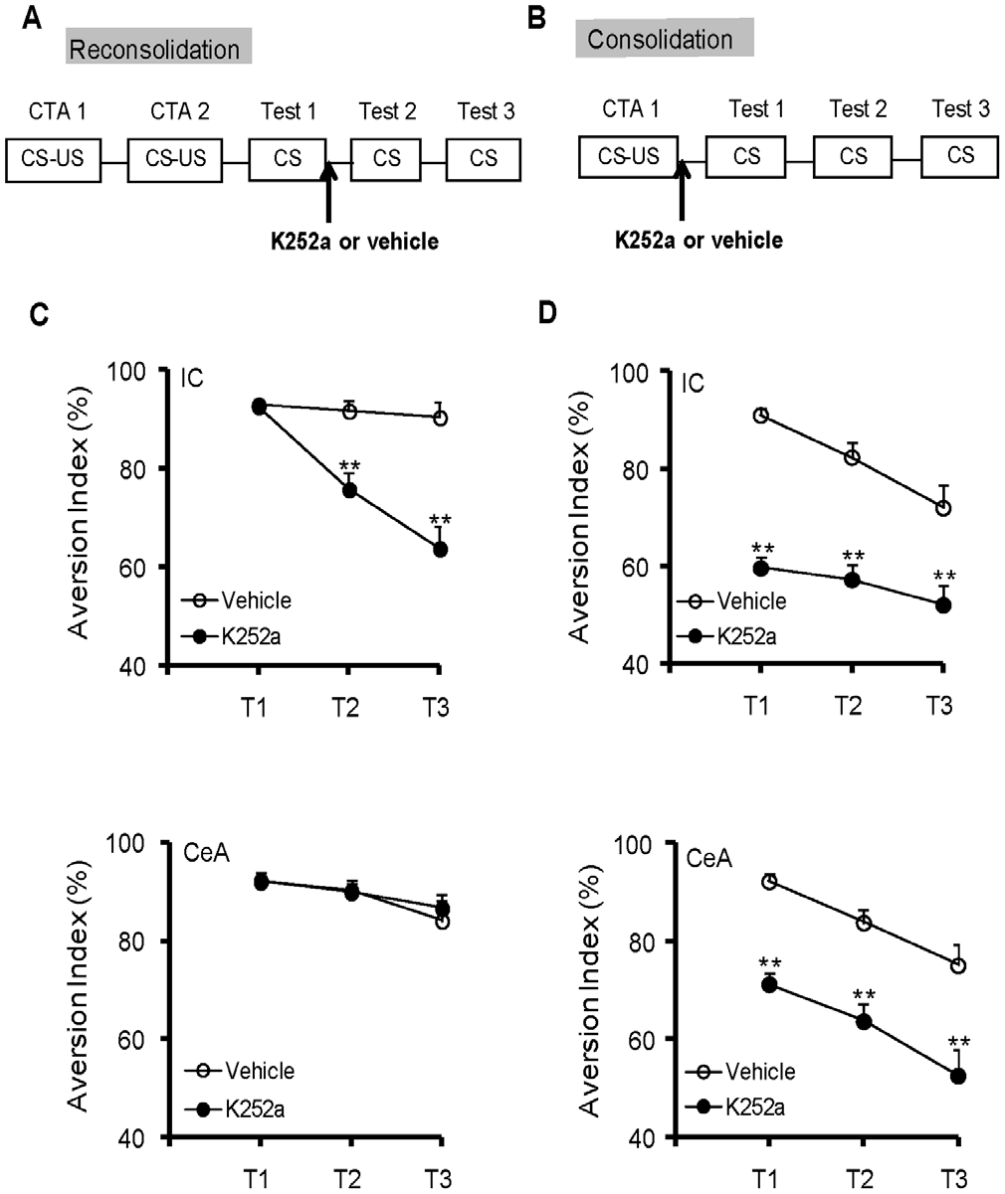
The microinjection of K252a into the IC or CeA could differentially impair CTA consolidation and reconsolidation. (A, B) Schematic of the K252a administration during CTA reconsolidation (A) or consolidation (B). Immediately after the first CTA test (reconsolidation) or CTA training (consolidation), the rats were treated with K252a. (C) The microinjection of K252a into the IC but not the CeA could disrupt the reconsolidation of CTA. n = 8–9 per group. **P<0.01, compared with the AIs in the vehicle group at different test points. (D) The microinjection of K252a into the IC or CeA could disrupt the consolidation of CTA. n = 8–9 per group. **P<0.01, compared with the AIs in the vehicle group at different test points. All values are presented as the mean ± S.E.M.

### Surgery and Drug Microinjection

The surgery and microinjection procedure was performed according to previously published protocols [19]. In brief, all of the animals were deeply anesthetized with chloral hydrate (5% w/v, i.p.), and were then restrained in a stereotactic apparatus (Reward Life Science, 8001). The skull was exposed and the stainless guide cannulas (23 gauge) were implanted bilaterally into the target regions, aiming 1.0 mm above the IC or the CeA [IC, anteroposterior (AP)+1.2 mm, lateral (L) ±5.3 mm, and ventral (V) −5.5 mm; CeA, AP −2.2 mm, L ±4.0 mm, and V −7.4 mm. All coordinates are relative to the bregma (Paxinos and Watson, 1996)]. The cannulas were fixed in place with acrylic dental cement and anchored by skull screws; additionally, a stylus was inserted into each cannula to prevent clogging. At least 1 week was allowed for the rats to recover before the behavioral test.

For drug microinjection, the stylus was removed from the guide cannula, and a 28-gauge microinjection cannula (extending 1.0 mm from the tip) was inserted, which was attached via polyethylene tubing to a Hamilton microsyringe driven by an injection pump (KD Science, KDS310, US). K252a (Calbiochem, San Diego, CA; 25 µM, 1 µl/side) [24] diluted in artificial CSF (ACSF)/0.05% dimethyl sulfoxide (DMSO) was used in the experiment to block Trk receptors. BDNF oligonucleotides (ODNs) [sequences used as follows: BDNF antisense oligonucleotide (ASO): 5′ TCT TCC CCT TTT AAT GGT 3′; BDNF missense oligonucleotide (MSO): 5′ ATA CTT TCT GTT CTT GCC 3′, 2 nM, 0.5 µl/side] were dissolved in sterile saline [10], [19]. The ASO was used to inhibit BDNF synthesis, while the MSO served as the control. Both BDNF ODNs were HPLC-purified phosphorothioate end-capped 18-mer sequences, which were relatively stable and less toxic. Human recombinant BDNF (hrBDNF, 0.25 µg/µl, 1 µl/side, PeproTech), prepared in sterile saline, was used for the rescue experiment [19], [25].

**Figure 3:**
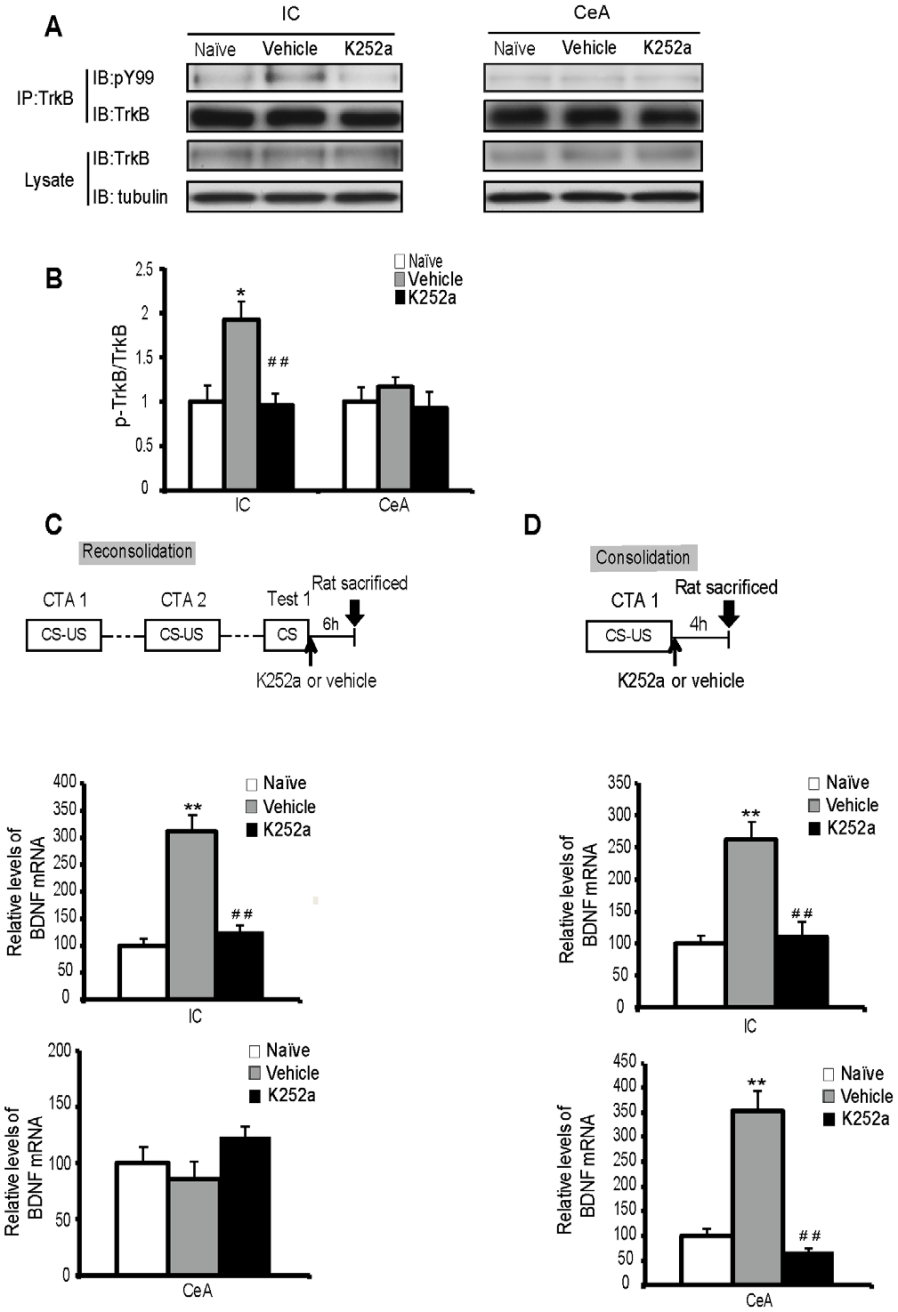
Treatment with K252a impaired CTA reconsolidation and consolidation through inhibition of the BDNF signal pathway and an increase in BDNF expression. (A, B) During CTA reconsolidation, the microinjection of K252a into the IC effectively blocks signal transduction through the TrkB receptors. The samples were obtained at 4 h post retrieval. The quantitation of the immunoblotting in (A) is shown as a percentage of the p-TrkB receptor relative to the total TrkB obtained from immunoprecipitation, which was normalized to the naïve group. n = 3 per group. *P<0.05, compared with the values of the naïve group; ^##^P<0.01, compared with the values of the vehicle-treated group. (C) The microinjection of K252a into the IC but not the CeA could block the increase of BDNF mRNA through the reconsolidation of CTA. (D) K252a microinjection into the IC or CeA could block the increase of BDNF mRNA induced through the consolidation of CTA. n = 5–6 per time point. **P<0.01, compared with the naïve group. ^##^ *P*<0.01, compared with the vehicle-treated group. All values are presented as the mean ± S.E.M.

### RT-PCR

Quantitative real-time PCR was used to determine the changes in BDNF mRNA levels. Total RNA was first prepared from the IC or CeA using TRIzol-A^+^RNA isolation reagent (TIANGEN, DP421-02, Shanghai). A 0.5 µg aliquot of each sample was treated with DNase to avoid DNA contamination and was then reverse-transcribed using the RevertAid First Strand cDNA Synthesis Kit (Fermentas, K1621, CA). The single-band primer sequences for BDNF were as follows: forward primer, 5′ TAA ATG AAG TTT ATA CAG TAC AGT GGT TCT ACA 3′; and reverse primer, 5′ AGT TGT GCG CAA ATG ACT GTT T 3′, β-actin was selected as the reference, and its primer sequences were as follows: forward primer, 5′ TCC ATC ATG AAG TGT GAC GT 3′; and reverse primer, 5′ GAG CAA TGA TCT TGA TCT TCA T 3′. Quantitative real-time PCR was performed using a Light Cycler 2.0 (Roche, Switzerland) with SYBR green chemistry (Takara, DRR041A, Dalian). Using the standard curve method, all primer efficiencies were evaluated; only the primers with amplification efficiencies between 90% and 110% were selected. Moreover, melting curve (dissociation curve) analysis was performed after the real-time PCR to ensure that the desired amplicon was detected. The threshold cycle for each sample was chosen from the linear range and was converted to a starting quantity by interpolation from a standard curve run on the same plate for each set of primers. Each sample was assayed in triplicate, and the mRNA levels were normalized for each well to the β-actin mRNA levels using the 2^−ΔΔCT^ method. A no-reverse-transcriptase control was included during the reverse transcription step to determine if an RNA sample was contaminated with genomic DNA.

### Histology

After the behavioral experiments were completed, the rats were deeply anesthetized, and 1 µl of methylene blue was microinfused. The rats were then perfused with saline followed by 4% paraformaldehyde in PBS. Brain sections (60 µm) were analyzed to verify the microinfusion sites. Only rats with the microinfusion sites and the scope of solution diffusion within the boundaries of the IC or the CeA were included in the data analysis.

**Figure 4:**
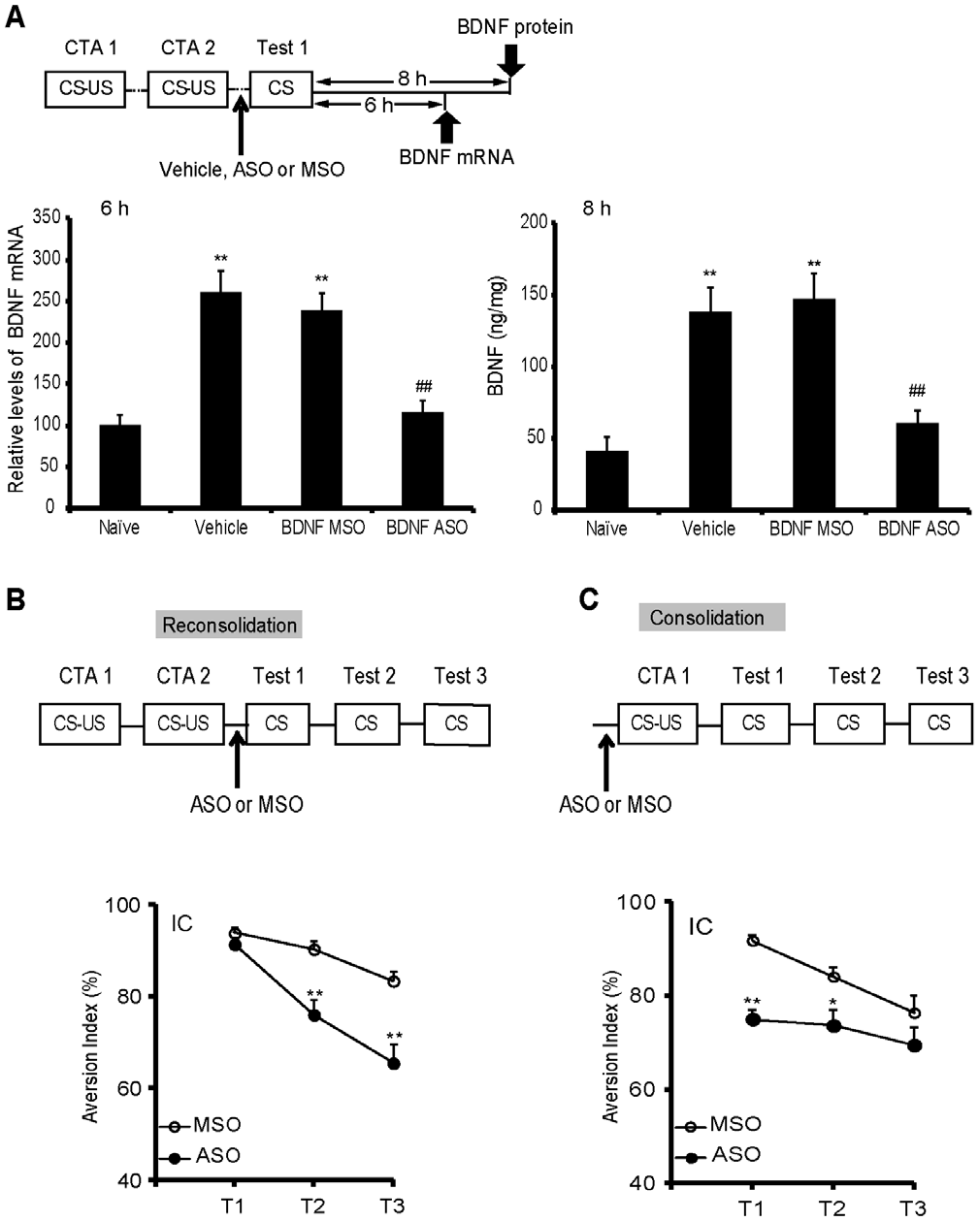
BDNF synthesis in the IC is necessary for both CTA reconsolidation and consolidation. (A) BDNF ASO treatment could block the increases in BDNF gene expression and protein synthesis levels in the IC induced by CTA reconsolidation. Intracerebral injections were administered at 90 min before retrieval, and the rats were sacrificed at 6 or 8 h post retrieval to detect BDNF mRNA or BDNF protein. n = 5–6 per group. **P<0.01, compared with the naïve group. ^##^ *P*<0.01, compared with the BDNF MSO-treated group. (B) BDNF ASO microinjection into the IC before retrieval significantly impairs CTA memory in the second and third test. (C) The microinjection of BDNF ASO into the IC before CTA conditioning could disrupt the formation of CTA. n = 8–9 per group. *P<0.05, **P<0.01, compared with the AIs in the MSO group at the respective test points. All values are presented as the mean ± S.E.M.

### Immunoprecipitation and Immunoblotting

The TrkB phosphorylation (p-TrkB) analysis method described in this study has been used in previous studies in our laboratory [19]. In brief, the rats in the different groups were killed 4 h after the first CTA test. The IC were dissected and homogenized in ice-cold lysis buffer containing 137 mM NaCl, 10 mM Tris–HCl, pH 8.0, 1 mM EDTA, pH 8.0, 1% NP-40, 10% glycerol, 1 mM phenylmethylsulphonyl fluoride, 10 mg/ml aprotinin, 1 mg/ml leupeptin and 0.5 mM sodium vanadate. The tissue homogenate solutions were centrifuged at 14,000×g for 5 min at 4°C. The supernatants were collected, and the protein concentrations were determined using BCA reagent (Thermo Scientific). Five milligrams of homogenized lysate was immunoprecipitated using rabbit anti-TrkB antibodies (1∶100, Millipore) followed by immunoblotting with mouse anti-phospho-tyrosine antibodies pY99 (1∶4000, Santa Cruz Biotechnology) and mouse anti-TrkB antibodies (1∶1000, BD Transduction Laboratories). For total TrkB analysis, the homogenized brain lysate was immunoblotted with mouse anti-TrkB antibodies (1∶1000, BD Transduction Laboratories) and mouse anti-tubulin (1∶10,000, Sigma-Aldrich) antibodies. For densitometric analyses, the immunoreactive bands were scanned and analyzed with MetaMorph software (Molecular Devices). The ratios of the immunoprecipitated p-TrkB to the total TrkB derived from the control groups were normalized to 1.0. The values of experimental groups were normalized according to their respective control groups.

### ELISA

To study the BDNF protein changes in different brain areas during reconsolidation, the IC and the CeA were dissected at various time points after the first CTA test (n = 5 per time point) and were stored at −80°C until analysis. The brain tissue samples were homogenized as described in the procedure for immunoprecipitation and immunoblotting. The tissue homogenate solutions were centrifuged at 14,000×g for 5 min at 4°C. The supernatants were collected and used for quantification of the total protein using BCA reagent (Thermo Scientific) and BDNF levels, which were determined using the commercially available BDNF Emax Immunoassay System (BDNF Emax® ImmunoAssay System, Promega, USA). The ELISA was performed according to the manufacturer’s protocol. Briefly, 96-well plates were coated with anti-BDNF monoclonal antibody overnight at 4°C. The wells were then blocked with blocking buffer and incubated with samples and standards for 2 h at room temperature. Anti-human BDNF polyclonal antibody was used as a reporter antibody, and anti-IgY-horseradish peroxidase conjugate was used to detect the amount of specifically bound polyclonal antibody. After incubation with a chromagenic substrate, the color change was measured in an ELISA plate reader at 450 nm (Model 680 microplate reader, Bio-Rad Laboratories Ltd, CA). All samples were assayed in duplicate.

**Figure 5:**
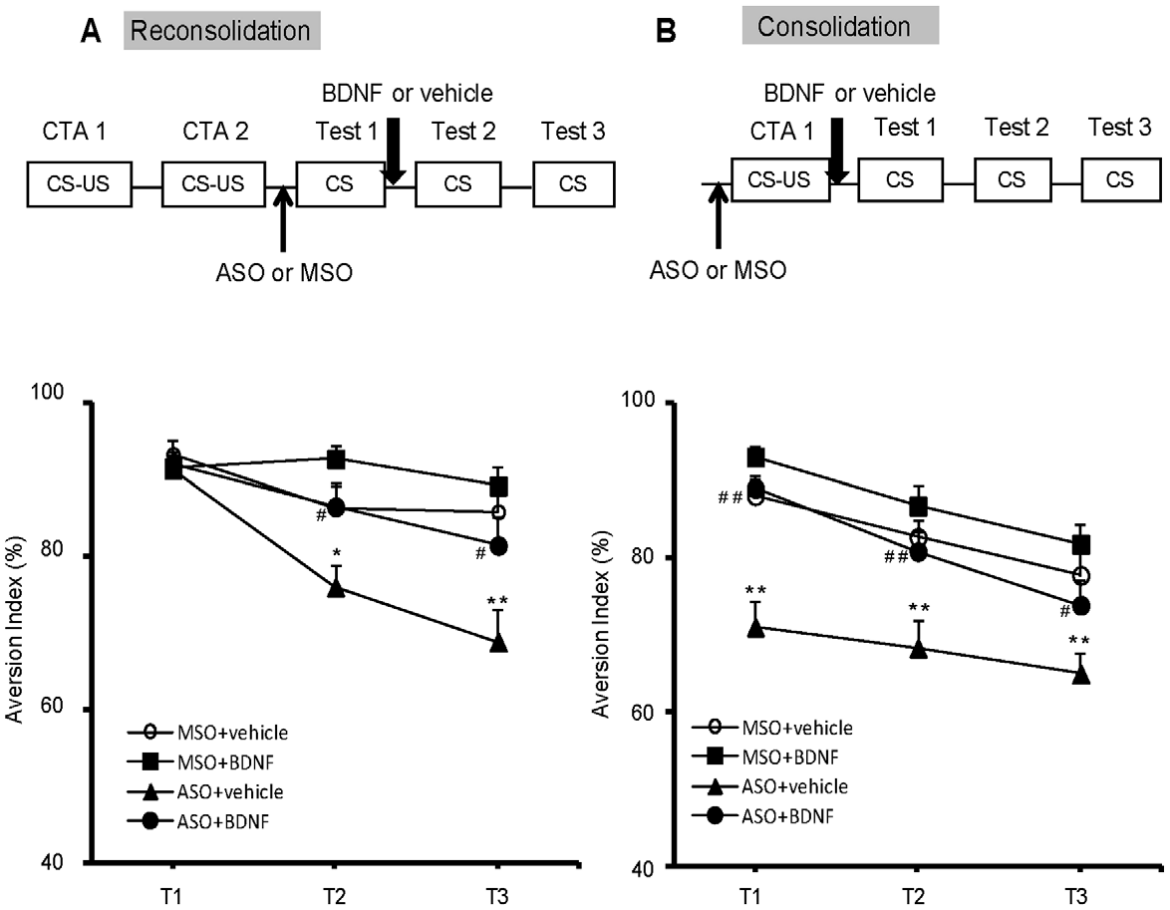
The exogenous administration of BDNF could rescue CTA reconsolidation and consolidation deficits induced by BDNF ASO microinjection. (A) The exogenous BDNF microinjection after CTA retrieval could rescue the impaired memory caused by BDNF ASO. BDNF ODNs were microinjected into the IC at 90 min before the first CTA test, and hrBDNF or vehicle was injected into the IC immediately after CTA retrieval. (B) The exogenous BDNF microinjection after CTA conditioning could rescue the memory deficit caused by BDNF ASO. n = 8–9 per group. *P<0.05, **P<0.01, compared with the MSO+vehicle group at the respective test points.^ #^ *P*<0.05, ^##^ *P*<0.01, compared with the ASO+vehicle-treated group. All values are presented as the mean ± S.E.M.

### Data Analyses

All of the data were statistically analyzed using the independent samples *t*-test or one-way ANOVA, followed by *post hoc* comparisons. A significance level of 0.05 was used for all analyses, and the statistical values in the text represented the means ± S.E.M. The statistical analysis software SPSS (Ver. 13.0) was employed.

**Figure 6:**
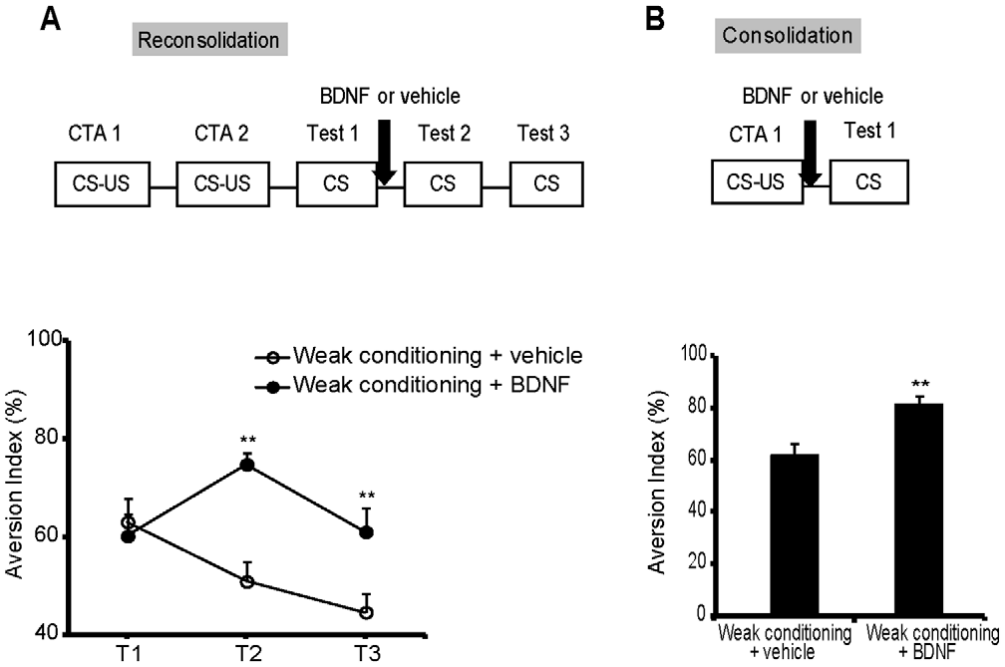
Exogenous BDNF microinjection into the IC after CTA retrieval or conditioning could enhance CTA memory. (A) The exogenous microinjection of BDNF after CTA retrieval could enhance the attenuated memory caused by weak conditioning. hrBDNF or vehicle was injected into the IC immediately after CTA retrieval. (B) The exogenous BDNF microinjection after CTA conditioning could enhance the attenuated memory caused by weak conditioning. n = 8 per group. **P<0.01, compared with the weak conditioning+vehicle group. All values are presented as the mean ± S.E.M.

## Results

### BDNF Levels Change in the IC and CeA during CTA Reconsolidation

According to previous reports, single trial CTA training is suitable for investigating the acquisition, consolidation and extinction of CTA [21], [22]. However, to detect reconsolidation in CTA, intensified CTA training with double trials is necessary to avoid the extinction trace (i.e., the inhibitory, CS-no US trace), which dominates the control of the rat’s behavior after the retrieval test [21], [22]. For this reason, all experiments for reconsolidation were conducted with an additional trial of CTA training compared with that of consolidation. We previously observed that BDNF mRNA levels in both the IC and CeA increased after CTA acquisition and showed that BDNF synthesis in these two areas is required for CTA consolidation [19]. In the present study, we attempted to determine whether the expression of BDNF in the IC and CeA is involved in CTA reconsolidation. Therefore, we first monitored the BDNF mRNA expression in the IC and CeA using real-time PCR at various time points after retrieval (Test 1), which triggered CTA reconsolidation (Fig. 1A). As shown in Fig. 1B, we observed significant changes in the BDNF mRNA levels during CTA reconsolidation in the IC [*F*_(5, 29)_ = 5.605, *P = *0.001] but not in the CeA [*F*_(5, 24)_ = 0.694, *P = *0.633]. The BDNF mRNA levels in the IC increased at 2 h (*post hoc*, LSD, *P = *0.048) and peaked at 6 h (*post hoc*, LSD, *P<*0.001) after retrieval to trigger reconsolidation. To further establish that the changes in the BDNF mRNA levels in the IC were specifically induced through the process of memory reconsolidation, a no reconsolidation group was subjected to double trial CTA training and no retrieval on the test day. As shown in Fig. 1C, at 6 h after retrieval, the BDNF mRNA levels in the IC significantly increased only in the reconsolidation group but not in the naïve and no reconsolidation groups [*F*_(2,12)_ = 15.648, *P*<0.001]. Moreover, there were no significant differences in the BDNF mRNA levels in the CeA among these three groups [*F*_(2,12)_ = 0.597, *P* = 0.566]. These results suggested that there were temporal- and spatial-specific changes in BDNF gene expression during CTA reconditioning, which was different from during consolidation.

Changes in the BDNF protein levels during CTA reconsolidation were also investigated. At various time points after the CTA test, the IC and CeA were dissected, and the BDNF protein levels were measured using ELISA. Consistent with changes in the BDNF mRNA levels, the BDNF protein levels in the IC [Fig. 1D; *F*_(5,24)_ = 5.121, *P* = 0.002] but not in the CeA [Fig. 1D; *F*_(5,24)_ = 0.809, *P* = 0.56] were significantly increased during CTA reconsolidation. The BDNF protein levels in the IC increased at 4 h (*post hoc*, LSD, *P* = 0.02), peaked at 8 h (*post hoc*, LSD, *P* = 0.001) and returned to baseline at 12 h (*post hoc*, LSD, *P* = 0.094) after retrieval, suggesting that the altered protein synthesis was associated with an increase in gene transcription.

The biological functions of BDNF are primarily mediated through the TrkB receptor. BDNF binds and activates the TrkB receptor through the dimerization and autophosphorylation of tyrosine residues in the intracellular domain of the TrkB receptor. To determine whether increased BDNF levels could lead to TrkB activation, we evaluated the p-TrkB levels using immunoprecipitation with the TrkB antibody and immunoblotting with the phosphotyrosine antibody (Fig. 1E). The results of the quantitative densitometric analyses showed elevated levels of p-TrkB in the IC [Fig. 1F; *t*_(4)_ = −3.540, *P* = 0.024] but not in the CeA [Fig. 1F; *t*_(4)_ = −0.847, *P* = 0.445] at 4 h post retrieval. Together, these results suggested that BDNF in the IC but not in the CeA might play a role in CTA reconsolidation through TrkB activation, which was different from the mechanism of consolidation.

### Blockade of BDNF Signaling in the IC and CeA by K252a Differentially Impairs CTA Consolidation and Reconsolidation

Although the previous experiment showed temporal and spatial changes in the BDNF expression in the IC during CTA reconsolidation, it is still unknown whether the increased BDNF level is functionally necessary for CTA reconsolidation. To address this question, the Trk receptor inhibitor K252a was utilized to block BDNF/TrkB signaling during reconsolidation (Fig. 2A). The histological verification of the drug injection sites is shown in Figure S2. Compared with the vehicle group, the K252a group showed similar AIs in the first test [Fig. 2C; IC, *t*_(14)_ = 0.157, *P* = 0.877; CeA, *t*_(15)_ = 0.153, *P* = 0.881], which suggested that these groups obtained an identical aversive memory. K252a was subsequently microinjected into the IC or CeA after the first test, which initiated the reconsolidation process. Compared with the AIs in the vehicle group, the AIs decreased significantly in the second and third test after K252a was administered into the IC [Fig. 2C; 2^nd^ test, *t*_(14)_ = 3.938, *P* = 0.001; 3^rd^ test, *t*_(14)_ = 5.033, *P*<0.001], suggesting that an inhibition of BDNF signaling in the IC disrupted CTA reconsolidation. By contrast, the microinfusion of K252a into the CeA during reconsolidation had no effect on the AIs measured during the next two days compared with the AIs of the vehicle group [Fig. 2C; 2^nd^ test, *t*_(14)_ = 0.015, *P* = 0.988; 3^rd^ test, *t*_(14)_ = −0.498, *P* = 0.626], suggesting that BDNF signaling in the CeA was not involved in the reconsolidation process of CTA memory.

To compare the role of BDNF in reconsolidation versus consolidation, we microinjected K252a into the IC or CeA after standard CTA training, which initiated memory consolidation (Fig. 2B). The rats receiving K252a microinjection into the IC or CeA showed decreased AIs during the first test compared with the rats in the vehicle groups [Fig. 2D; IC, *t*_(15)_ = 12.961, *P*<0.001; CeA, *t*_(14)_ = 7.891, *P*<0.001], suggesting that BDNF signaling in both the IC and CeA were required for CTA memory consolidation. During the next two days, the AIs of the K252a-injected rats were lower than the AIs of the vehicle-treated rats [IC, 2^nd^ test, *t*_(15)_ = 5.857, *P*<0.001; 3^rd^ test, *t*_(14)_ = 3.215, *P* = 0.006; CeA, 2^nd^ test, *t*_(14)_ = 4.779, *P*<0.001; 3^rd^ test, *t*_(14)_ = 3.4, *P = *0.004].

We also investigated whether K252a microinjection could indeed block BDNF signaling during reconsolidation. We observed, that the elevated p-TrkB levels at 4 h post retrieval were totally blocked upon K252a microinjection in the IC (Fig. 3A, B; *post hoc*, LSD, *P* = 0.009). However, the microinfusion of K252a into the CeA had no significant effect on the p-TrkB levels compared with levels in the vehicle-treated group (Fig. 3A, B; *post hoc*, LSD, *P* = 0.312). Moreover, K252a microinjection into the IC but not the CeA blocked the reconsolidation-induced increase in BDNF mRNA levels at 6 h post retrieval compared with the levels in the vehicle-treated group [Fig. 3C; *post hoc*, LSD; IC, *P<*0.001; CeA, *P = 0.074*]. For consolidation, according to our previous study [19], the BDNF mRNA levels in the IC or CeA peaked at 4 h after conditioning. Therefore, we selected 4 h post conditioning time point to measure the BDNF mRNA levels during consolidation. We observed that the K252 administration into either the IC or CeA completely blocked the elevation of BDNF mRNA levels through consolidation (Fig. 3D; *post hoc*, LSD; IC, *P*<0.001; CeA, *P*<0.001). Altogether, these results suggested that BDNF signaling in the IC and CeA played different roles during reconsolidation and consolidation.

### BDNF Synthesis in the IC is Required for Both CTA Reconsolidation and Consolidation

Because K252a is a pharmacological inhibitor of Trk receptors and might have nonspecific effects, BDNF ASO was further used to specifically investigate the role of newly synthesized BDNF in CTA memory reconsolidation and consolidation [19]. The K252a studies showed that BDNF signaling in the IC but not in the CeA might be involved in CTA reconsolidation; therefore, we selected the IC for further study using BDNF ASO. According to the previous report [10], ODNs generally require a long time to exert their effects, so we microinjected BDNF ODNs into the IC at 90 min before the first test for reconsolidation or 90 min before CS-US coupling for consolidation (Fig. 4B, C). We first examined the effects of BDNF ASO on BDNF mRNA and protein levels during reconsolidation (Fig. 4A). Vehicle and BDNF MSO, which included the same 18 nt as the ASO but in a scrambled order, served as controls. Although the mRNA and protein levels of BDNF in the vehicle- or BDNF MSO-treated group increased significantly when compared with the naïve group (p*ost hoc*, LSD; all *P*<0.001), the increased values indicated no differences between these two groups (*post hoc*, LSD; mRNA, *P* = 0.423; protein, *P* = 0.640), suggesting the effects on BDNF mRNA and protein were related to the process of reconsolidation but not the MSO. Moreover, compared with the MSO group, the group with BDNF ASO microinfusion in the IC at 90 min before retrieval had BDNF mRNA (*post hoc*, LSD, *P*<0.001) and protein levels (*post hoc*, LSD, *P* = 0.001) that were blocked during reconsolidation, which demonstrated that BDNF ASO could efficiently block BDNF synthesis. The behavior results showed that, compared with the MSO group, the group with the microinfusion BDNF ASO into the IC before retrieval had significantly decreased AIs during the second [*t*_(15)_ = 4.109, *P* = 0.001] and third [*t*_(15)_ = 3.964, *P* = 0.001] tests; however, no effect was observed in the first test [*t*_(15)_ = 1.344, *P* = 0.199] (Fig. 4B). Compared with the MSO group, the group with the administration of BDNF ASO in the IC for consolidation before conditioning had significantly disrupted CTA memory during the first test [*t*_(16)_ = 6.970, *P*<0.001] (Fig. 4C). These data further supported the results of our previous K252a studies and suggested that BDNF synthesis in the IC played an important role in both CTA memory reconsolidation and consolidation.

### Exogenous BDNF could Rescue CTA Reconsolidation and Consolidation Deficits Induced by BDNF ASO Microinjection

Because the previous loss-of-function experiments showed that BDNF *de novo* synthesis in the IC is necessary for CTA reconsolidation and consolidation, we examined whether exogenous BDNF administration could rescue BDNF ASO microinjection-induced deficits in CTA reconsolidation and consolidation. For reconsolidation, exogenous BDNF or vehicle was microinjected into the IC after the administration of BDNF ASO or MSO (Fig. 5A). Compared with that of the ASO+vehicle-treated group, the AIs of the rats in the ASO+BDNF-treated group significantly increased in tests conducted for the next two days (*post hoc*, LSD; 2^nd^ test, *P = *0.010; 3^rd^ test, *P* = 0.012), suggesting that the application of exogenous BDNF could rescue the BDNF ASO-induced deficit in CTA reconsolidation. The results also showed that although the AIs in MSO+BDNF-treated rats increased, this result did not reach statistical significance compared with AIs of the MSO+vehicle group (*post hoc*, LSD; 2^nd^ test, *P = *0.107; 3^rd^ test, *P* = 0.481), which might reflect the ceiling effect of the high AIs values. Thus, a weak CS-US conditioning protocol was used in the next experiment to investigate the role of BDNF alone on CTA reconsolidation.

For consolidation, exogenous BDNF introduced into the IC after conditioning rescued the attenuated CTA memory caused by BDNF ASO administration in the first test (Fig. 5B; 1^st^ test, *post hoc, LSD, P<*0.001, versus ASO+vehicle group). However, compared with the MSO+vehicle treatment, the MSO+exogenous BDNF treatment did not further increase the AIs (1^st^ test, *post hoc, LSD, P = *0.146), which might also reflect the ceiling effect. These results suggested that introducing exogenous BDNF into the IC may effectively rescue the deficit in CTA memory reconsolidation and consolidation caused by BDNF ASO.

### Exogenous BDNF could Enhance CTA Memory Reconsolidation and Consolidation

To investigate whether BDNF alone plays a role in CTA reconsolidation and consolidation and avoid the “ceiling effect” of high AIs, a weak CTA training protocol in which rats were injected with a low concentration of LiCl (0.075 M) was conducted. Previous reports have shown that weak CTA training would result in an attenuated CTA memory [19]. Compared with the vehicle group, the group with the administration of BDNF in the IC during CTA reconsolidation had significantly increased AIs during the second and third tests (Fig. 6A; *t _(11.18)_’ = *−*5.042*, *P*<0.001). Compared with the vehicle group, the group that had exogenous BDNF infusion into the IC for consolidation had enhanced CTA memory (Fig. 6B; *t _(8.95)_’ = 5.171*, *P* = 0.001). These results suggested that BDNF in the IC is sufficient for CTA reconsolidation and consolidation.

## Discussion

The aim of our study was to investigate the role of BDNF in CTA reconsolidation and consolidation. Our evidence suggested that increased BDNF synthesis in the IC but not in the CeA was temporally induced during CTA reconsolidation. BDNF in the IC is functionally required for CTA reconsolidation, whereas BDNF signaling in both the IC and CeA is necessary for CTA consolidation. Moreover, we observed that the exogenous administration of BDNF into the IC could not only rescue the reconsolidation deficit from the inhibition of BDNF synthesis but also enhance the attenuated memory induced through weak CTA training.

While CTA consolidation is well studied and well understood, few studies with contradictory results have elucidated the mechanisms underlying CTA reconsolidation. Our data provide several new insights into CTA reconsolidation and consolidation. First, we observed increased BDNF mRNA expression and synthesis in the IC not only during CTA consolidation but also during reconsolidation. Moreover, the newly synthesized BDNF in the IC activates TrkB receptors. To the best of our knowledge, these results are the first to show that BDNF is involved in CTA reconsolidation. Previous reports that use the contextual fear conditioning paradigm have shown that BDNF in the hippocampus is not involved in memory reconsolidation [10]. We hypothesized that the discrepancy might reflect a different memory paradigm and brain region. Interestingly, we also observed that the injection of K252a into the IC, which impeded BDNF/TrkB signaling, blocked the increase in BDNF mRNA levels during reconsolidation, suggesting that TrkB activation induces a signaling cascade to promote BDNF synthesis during BDNF reconsolidation.

Second, we observed that BDNF in the IC was the cellular substrate for both consolidation and reconsolidation, while BDNF in the CeA was specifically involved in CTA consolidation but not reconsolidation. The neural circuit of CTA memory may be associated with the pathways for gustatory and visceral aversive stimuli. Using lesion studies, some areas in these pathways, including IC and CeA, had been shown to be involved in CTA memory [26]. Moreover, some neurotransmitters (e.g., those that belong to the cholinergic system or glutamatergic system) and cellular processes (e.g., Ras–MAP kinase signaling pathway, CREB phosphorylation, protein synthesis, BDNF) in these areas play important roles in CTA consolidation [19], [26]. However, the administration of protein synthesis inhibitors for CTA reconsolidation suggested the involvement of the IC and CeA [21], [27], but the detailed molecular mechanism underlying reconsolidation in these areas are still unknown. In this study, K252a and BDNF ASO blocked BDNF signaling and *de novo* synthesis, respectively, and we observed that BDNF in the IC, but not in the CeA, is functionally necessary for CTA memory reconsolidation. While most studies of reconsolidation were focused on consolidation, and the molecular mechanism and cellular substrates of consolidation and reconsolidation were largely shared, several studies have reported dissociations between these processes for particular plasticity molecules or for plasticity in general within certain brain regions [5], [10], [28], [29], [30], [31]. Our study further demonstrated that BDNF signaling in the IC and CeA played different roles in memory reconsolidation and consolidation. As mentioned, reconsolidation is a process that incorporates new information into a previously consolidated trace [4], [27], and the IC is the primary area for CTA memory storage [32]. These specific characteristics for reconsolidation might provide an explanation for the observed differences between reconsolidation and consolidation.

Finally, we showed that exogenous BDNF administration in the IC could not only rescue the CTA memory reconsolidation deficit induced by BDNF ASO injection but also enhance the attenuated memory induced by weak CTA training. These results suggested that BDNF in the IC is necessary and sufficient for CTA reconsolidation. The pharmacological manipulation of BDNF expression or signaling in the IC during reconsolidation might have clinical implications for memory disorders. For post-traumatic stress disorder patients, combining exposure therapy, which reactivates the trauma memories during reconsolidation, with the administration of the TrkB antagonist might be a potential effective therapeutic approach. In Alzheimer’s disease, the cognitive dysfunction is exacerbated by deficient reconsolidation [33], in which case the TrkB agonist or small molecules for the induction of BDNF expression might be effective to enhance memory and cognitive function.

In conclusion, using the CTA memory paradigm, we determined that BDNF in the IC but not in the CeA involves memory reconsolidation, while BDNF in both the IC and the CeA are required for memory consolidation. The differential spatial-specific roles of BDNF in memory consolidation and reconsolidation might provide a novel target for separately manipulating newly encoded and reactivated memories without causing universal amnesia.

## Supporting Information

Figure S1 **Schematic of CTA consolidation and reconsolidation.**
(TIF)

Figure S2 **Representative schematic of cannula tip localization in different brain regions.** (A) Representative schematic of cannula tip localization in the IC. (B) Representative schematic of cannula tip localization in the CeA.
(TIF)
