# Supplementary figures and images for: Differential Involvement of Brain-Derived Neurotrophic Factor in Reconsolidation and Consolidation of Conditioned Taste Aversion Memory
Source: PLoS One. 2012 Nov 21;7(11):e49942. doi: 10.1371/journal.pone.0049942 (PMC3503816; doi:10.1371/journal.pone.0049942)

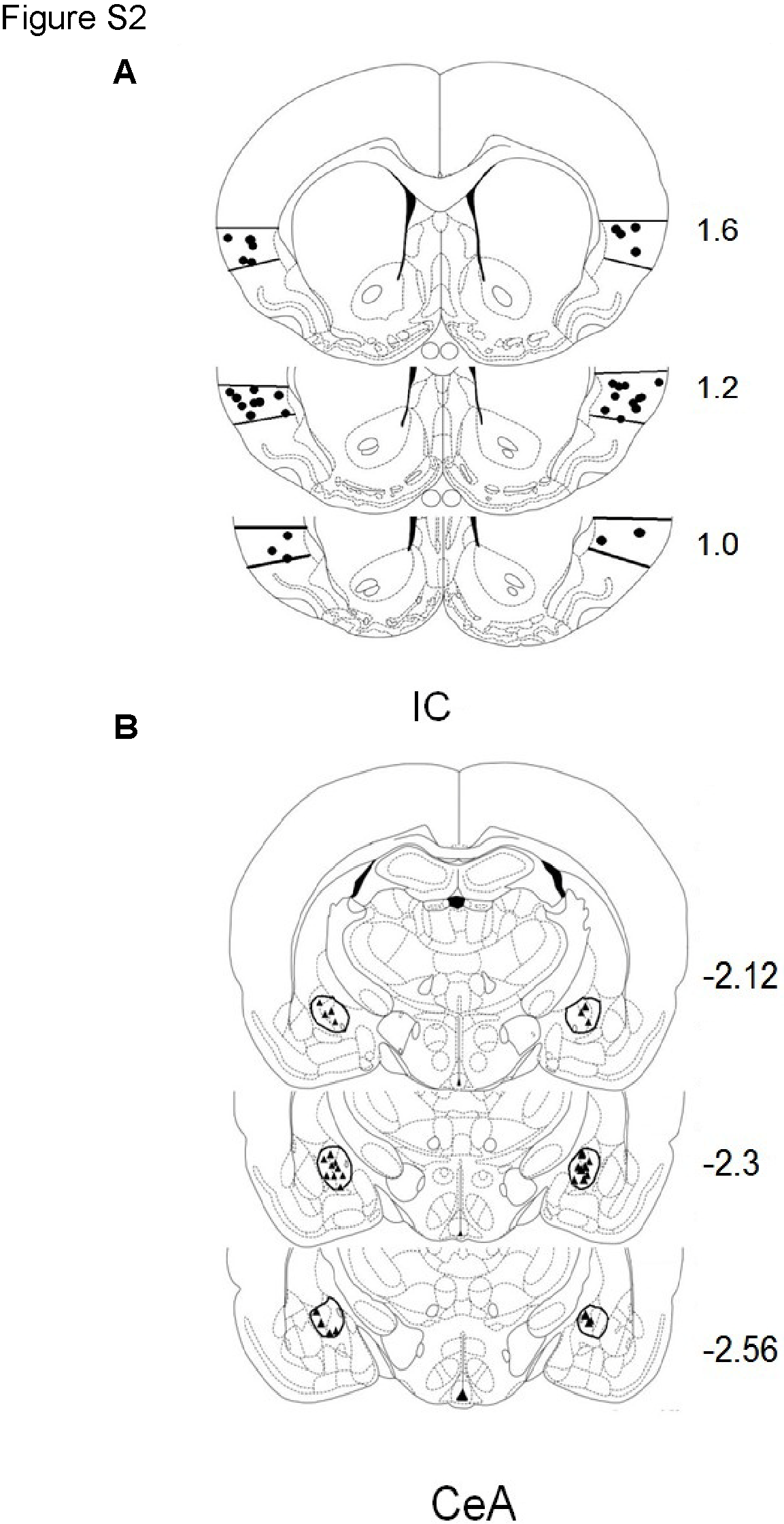

Supplement: Figure S2 — Representative schematic of cannula tip localization in different brain regions. (A) Representative schematic of cannula tip localization in the IC. (B) Representative schematic of cannula tip localization in the CeA. (TIF) [file pone.0049942.s002.tif]
